# Diagnostic utilities of washout CYFRA 21-1 combined with washout thyroglobulin for metastatic lymph nodes in thyroid cancer: a prospective study

**DOI:** 10.1038/s41598-024-58093-9

**Published:** 2024-03-31

**Authors:** Joonseon Park, Solji An, Kwangsoon Kim, Jeong Soo Kim, Chan Kwon Jung, Ja Seong Bae

**Affiliations:** 1 Department of Surgery, College of Medicine, The Catholic University of Korea, 222 Banpo-daero, Seocho-gu, Seoul, 06591 Republic of Korea; 2 Department of Hospital Pathology, College of Medicine, The Catholic University of Korea, Seoul, Republic of Korea

**Keywords:** Thyroid cancer, CYFRA 21-1, FNAC, Thyroglobulin, Washout, Thyroid cancer, Diagnostic markers

## Abstract

Cervical lymph node (LN) metastasis is common in differentiated thyroid cancer (DTC). This study evaluated the utility of the washout CYFRA 21-1 level, combined with the thyroglobulin (Tg) concentration, in terms of diagnosis of LN metastasis. We prospectively enrolled 53 patients who underwent thyroid surgery to treat DTC with lateral cervical LN metastases. Preoperative ultrasound guided needle localization was used to surgical sampling of specific LNs during the operation. The intraoperative washout Tg and CYFRA 21-1 levels were measured in such LNs. The Tg and CYFRA 21-1 levels differed significantly between metastatic and benign LNs. The cutoff values were 2.63 ng/mL for washout CYFRA 21-1 and 22.62 ng/mL for Tg. Combined use of the washout Tg and CYFRA 21-1 levels afforded the highest diagnostic accuracy (92.5%), better than that of individual markers. The sensitivity, specificity, positive predictive value (PPV), negative predictive value (NPV) were 94.6%, 90.0%, 91.4%, 93.8%, respectively. The conjunction of the washout CYFRA21-1 and Tg levels enhances the diagnostic accuracy of LN metastasis in DTC patients. The washout CYFRA 21-1 level may be useful when malignancy is suspected, especially in cases where the cytology and washout Tg findings do not provide definitive results.

## Introduction

Differentiated thyroid cancer (DTC) typically exhibits a favorable prognosis with 10-year disease specific survival (DSS) about 97%^1^. However, cervical lymph node (LN) metastasis is commonly found in DTC^2^, and the metastatic cervical LNs as locoregional recurrences after the initial surgery are observed in approximately 30% of cases^3–5^. Therefore, surgeons should thoroughly evaluate cervical LN metastasis status during initial work-up to determine the extent of surgery; close monitoring is also required after the initial operation.

However, LN enlargement detected by ultrasound (US) during diagnostic workup or follow-up after surgery is sometimes attributed to a simple inflammatory reaction, making it challenging to differentiate it from malignancy^6–8^. Various methods including imaging studies and pathologic confirmation have being proposed for accurate diagnosis of metastatic LNs before and after thyroid cancer surgery^8–11^. US-guided fine needle aspiration (FNA) is being utilized as a diagnostic modality with higher specificity compared to imaging studies. However, sometimes, sampling of inappropriate LNs or small metastatic lesions can yield false-negative US-guided FNA cytology (FNAC) results^12^. In such cases, the specific thyroglobulin (Tg) levels in FNA washout fluid have served to support diagnosis^12,13^. However, previous studies have used different washout Tg cutoff values and no consensus has yet emerged^14–16^. Also, in some cases, the washout Tg value is low but malignancy is cytologically evident, or the washout Tg value is high but the cytology is benign. The incidence of false negatives, characterized by low washout Tg values despite malignant cytology, is reported to be approximately 6–50%. Conversely, the false positive rate, where high washout Tg values are observed alongside benign cytology, with subsequent pathology also confirming benignity, ranges from approximately 2.5–23%. Both of these rates represent significant proportions that cannot be disregarded^17–20^. When the washout Tg concentration is high but the cytology negative, it is necessary to identify additional tumor markers that support and enhance diagnostic specificity and sensitivity. Cytokeratin fragment 21-1 (CYFRA 21-1) is a soluble fragment of the cytokeratin 19 protein that serves as a biomarker of LN metastasis and cell metastatic potential^21,22^. It is a useful diagnostic marker not only of thyroid but also stomach and ovarian cancers, laryngeal squamous cell carcinomas, liver metastases of colorectal cancer, and LN metastases of lung cancer^23–29^. In thyroid cancer, most prior studies have focused on serum levels of this marker^21,22,30,31^; few works have assayed CYFRA 21-1 concentrations in metastatic LN washouts^27,32^. A previous multidisciplinary study in our institution found that a combination of washout CYFRA 21-1 and Tg levels, and FNAC, afforded high sensitivity of 98.8%, highlighting the diagnostic utility of CYFRA 21-1^27^. However, this previous study has compared preoperative FNAC results with the final pathologies of all acquired LNs; precise LN matching with those localized in preoperative US-guided procedures has not been conducted.

The utility of US-guided hook-needle localization in thyroid surgery accompanied by LN metastasis is well-established^33–35^. If malignancy in LNs is suspected before surgery, US-guided hook-needle localization can be performed preoperatively to accurately identify the LNs that need to be excised, ensuring that essential LNs are not missed during surgery. In our institution, we often use needle localization prior to Level 2 and 5 LN dissection, especially when visual identification might be challenging due to intricate locations or when dealing with small-sized metastatic LNs. This approach significantly enhances surgical precision.

In this study, we utilized needle localization to facilitate the accurate retrieval of washout Tg and CYFRA 21-1 from specific LNs, concurrently ensuring the thorough removal of metastatic LNs, thereby deriving two distinct benefits. To the best of our knowledge, no study has yet matched LNs analyzed via FNAC before surgery with their individual final pathologies. The aim of this study is to investigate the clinical significance of supplementing the assessment of LN metastasis with preoperative localization through the measurement of the novel tumor marker, CYFRA 21-1.

## Results

### Baseline clinicopathological characteristics

Table 1 lists the basic characteristics of the 53 patients. The mean age was 42.2 years and females predominated (69.8%). Most patients (86.8%) underwent total thyroidectomy with modified radical neck dissection (mRND); 13.2% of patients underwent mRND only upon reoperation. The majority, 52 patients (98.1%), were diagnosed with PTC, while one patient (1.9%) presented with FTC. The mean tumor size was 1.8 cm (range, 0.2–6.2 cm). The multifocality and bilaterality rates were 47.8% and 15.2%, respectively. Lymphatic, vascular, and perineural invasion were observed in 84.8%, 2.2%, and 4.3% of cases, respectively. BRAF^V600E^ positivity was observed in 92.3% of cases. The distribution of the T category was: T1, 28 patients (60.9%); T2, 5 (10.9%); T3a, 3 (6.5%); T3b, 9 (19.6%); and T4a, 1 (2.2%), respectively. Most patients were of TNM stage I (82.6%); 17.4% were of stage II.

**Table 1:** Baseline clinicopathologic characteristics of the study population.

Total 53 patients	
Age (years)	42.2 ± 13.8 (range, 16–75)
Male:female	1:2.3
Male	16 (30.2%)
Female	37 (69.8%)
Extent of operation	
TT + mRND	46 (86.8%)
Only mRND	7 (13.2%)
Type of carcinoma	
PTC	52 (98.1%)
FTC	1 (1.9%)
Tumor size (cm)	1.8 ± 1.4 (range, 0.2–6.2)
ETE	10/46 (21.7%)
Multifocality	22/46 (47.8%)
Bilaterality	7/46 (15.2%)
Lymphatic invasion	39/46 (84.8%)
Vascular invasion	1/46 (2.2%)
Perineural invasion	2/46 (4.3%)
BRAF^V600E^ positive	48/52 (92.3%)
TERT positive	1/46 (2.2%)
Harvested LNs	60.4 ± 29.9 (range, 20–153)
Positive LNs	13.5 ± 8.8 (range, 2–39)
T category (n = 46)	
T1/T2/T3a/T3b/T4a	28 (60.9%)/5 (10.9%)/3 (6.5%)/9 (19.6%)/1 (2.2%)
N category (n = 46)	
N1b	46 (100%)
TNM stage (n = 46)	
Stage I/II	38 (82.6%)/8 (17.4%)

Data are expressed as patient’s number(%), or mean ± SD.

*TT* total thyroidectomy, *mRND* modified radical neck dissection, *PTC* papillary thyroid cancer, *FTC* follicular thyroid cancer, ETE extrathyroidal extension, *LN* lymph node, *Tg* thyroglobulin, *T* tumor, *N* node, *M* metastasis.

### Washout Tg and washout CYFRA 21-1 levels by LN status

**Figure 1:**
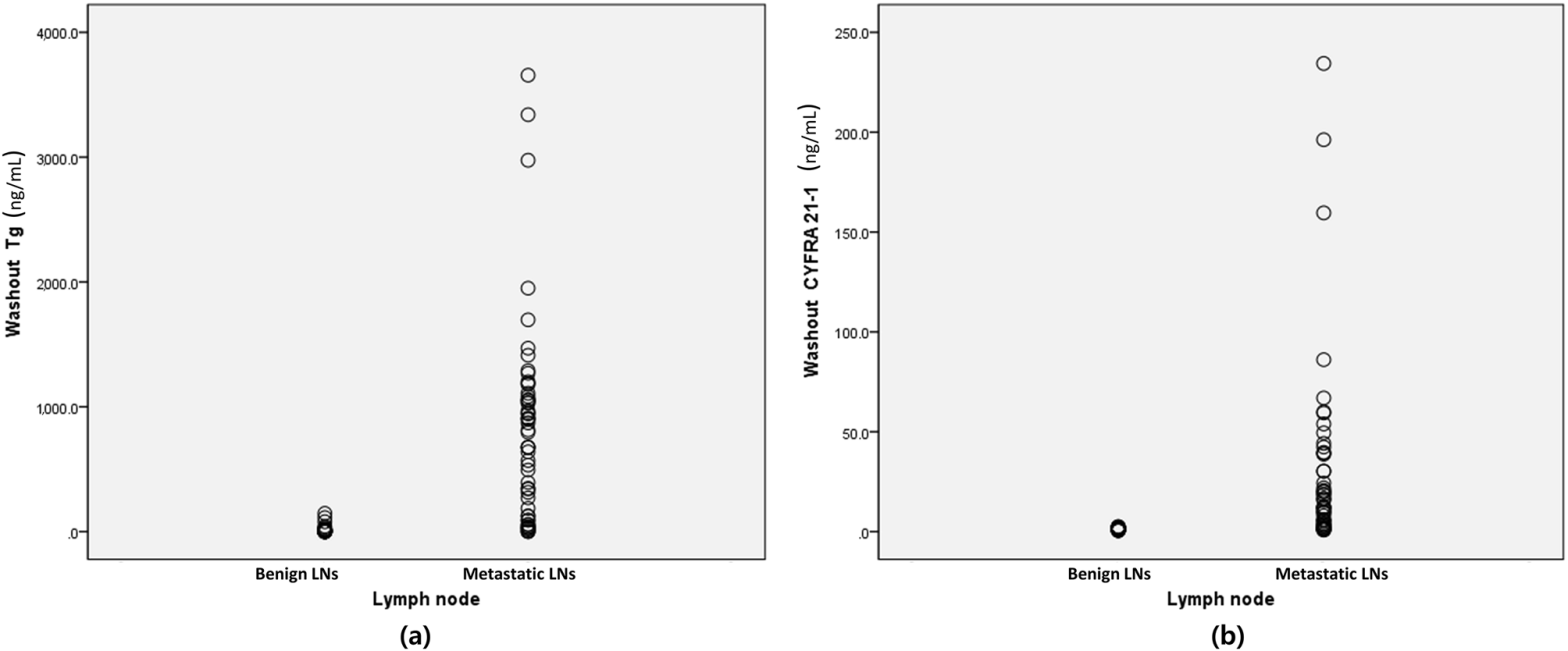
Scatterplots of measurements in benign and metastatic lymph nodes using fine-needle aspiration for (**a**) washout Tg and (**b**) washout CYFRA 21-1.

Both the washout Tg and washout CYFRA 21-1 levels differed significantly between metastatic and benign LNs (Fig. 1, Table 2). The mean washout Tg levels were 826.4 ± 794.3 ng/mL (range 0.06–3656.3 ng/mL) in metastatic LNs and 33.6 ± 145.4 ng/mL (range 0.01–1053.76 ng/mL) in benign LNs (*p* < 0.001). For washout CYFRA 21-1, the mean levels were 29.6 ± 46.4 ng/mL (range, 0.89–234.4 ng/mL) in metastatic LNs and 1.4 ± 0.8 ng/mL (range 0.45–5.56 ng/mL) in benign LNs (*p* < 0.001).

**Table 2:** Comparison of washout Tg and washout CYFRA 21-1 according to the status of lymph node.

	Metastatic LN	Benign LN	*p*-value
Tg (ng/mL)	826.4 ± 794.3 (range, 0.06–3656.3)	33.6 ± 145.4 (range, 0.01–1053.76)	< 0.001
CYFRA 21-1 (ng/mL)	29.6 ± 46.4 (range, 0.89–234.4)	1.4 ± 0.8 (range, 0.45–5.56)	< 0.001

Data are expressed as patient’s number (%), or mean ± SD.

A statistically significant difference was defined as *p* < 0.05.

*Tg* thyroglobulin, *LN* lymph node.

### Cutoff values and diagnostic capabilities of washout Tg and washout CYFRA 21-1

**Figure 2:**
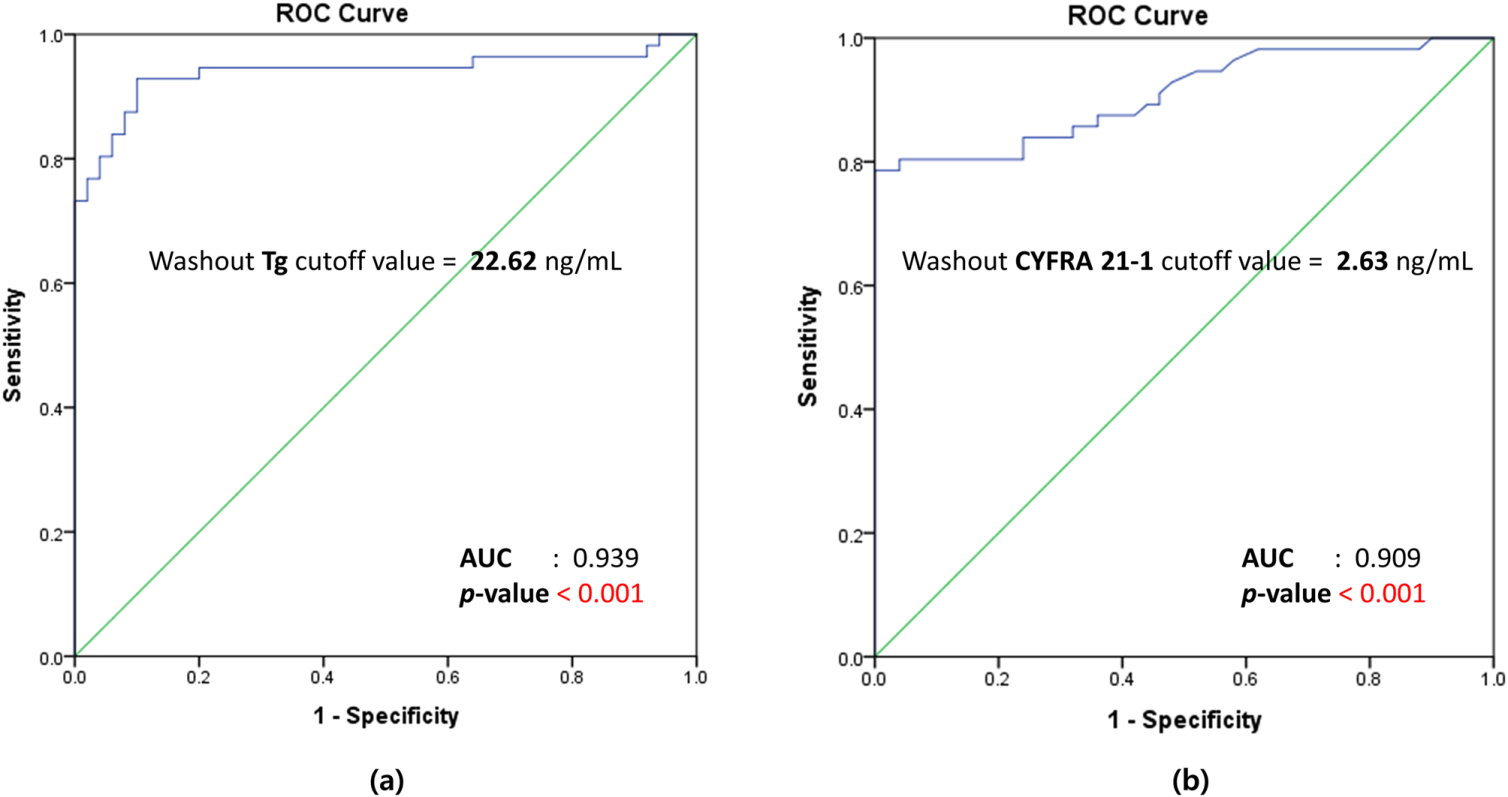
Receiver-operating characteristic (ROC) curves of (**a**) washout Tg and washout CYFRA 21-1. (**a**) The cutoff value for washout Tg was 22.62 ng/mL (AUC, 0.939; p < 0.001). (**b**) The cutoff value for washout CYFRA 21-1 was 2.63 ng/mL (AUC, 0.909; p < 0.001).

The results of receiver-operating characteristic (ROC) curve analyses for washout Tg and washout CYFRA 21-1 are shown in Fig. 2, and the diagnostic performances of the washout Tg and washout CYFRA 21-1 levels are presented in Table 3. The cutoff value for washout Tg that best predicted LN metastasis was 22.62 ng/mL (area under the curve (AUC), 0.939; sensitivity, 92.9%; specificity, 90.0%; PPV, 91.2%; NPV, 91.8%; accuracy, 91.5%; *p* < 0.001). The cutoff value for washout CYFRA 21-1 was 2.63 ng/mL (AUC, 0.909; sensitivity, 78.63%; specificity, 100.0%; PPV, 100.0%; NPV, 80.6%; accuracy, 88.7%; *p* < 0.001). When washout Tg and CYFRA 21-1 were combined, sensitivity was 94.6%, specificity was 90.0%, PPV was 91.4%, NPV was 93.8%, and accuracy was 92.5%, demonstrating the highest accuracy compared to measuring washout Tg or CYFRA 21-1 alone.

**Table 3:** Diagnostic comparison: washout Tg, washout CYFRA 21-1, and conjunction of Washout Tg & CYFRA 21-1.

	Sensitivity (%)	Specificity (%)	PPV (%)	NPV (%)	Accuracy (%)
Tg ≥ 22.62 ng/mL	92.9	90.0	91.2	91.8	91.5
CYFRA 21-1 ≥ 2.63 ng/mL	78.6	100.0	100.0	80.6	88.7
Tg + CYFRA 21-1	94.6	90.0	91.4	93.8	92.5

*Tg* thyroglobulin, *PPV* positive predictive value, *NPV* negative predictive value.

### The efficacy of washout Tg and washout CYFRA 21-1 according to clinical characteristics

Tables 4, 5, and 6 compared the diagnostic performance based on the combination criteria of Tg ≥ 22.62 ng/ml or CYFRA 21-1 ≥ 2.63 ng/ml, relative to the clinical characteristics of patients. While the diagnostic performance showed a tendency to be higher in the male group, younger age group, and aggressive histology group, these differences were not statistically significant. Table 4 demonstrates the diagnostic performance by gender, showing that this diagnostic tool overall exhibited superior performance in male patients compared to female patients (sensitivity, 100.0% vs. 92.7%; specificity, 94.1% vs. 87.9%; PPV, 93.8% vs. 90.5%; NPV, 100.0% vs. 90.6%; accuracy, 96.9% vs. 90.5%, respectively). Table 5 displayed the diagnostic performance of this test by age, indicating that the diagnostic performance was superior in the younger group (under 55 years) compared to the older group (sensitivity, 97.8% vs. 81.8%; specificity, 92.3% vs. 81.8%; PPV, 93.6% vs. 81.8%; NPV, 97.3% vs. 81.8%; accuracy, 95.2% vs. 81.8%, respectively). Table 6 compared the diagnostic performance of this test according to the aggressiveness of histology. In this study, non-aggressive histology included classic and follicular variants, while aggressive histology encompassed tall cell and diffuse sclerosing variants. The diagnostic performance of the test was superior in cases of aggressive histology compared to non-aggressive histology (sensitivity, 100.0% vs. 93.6%; specificity, 100.0% vs. 88.4%; PPV, 100.0% vs. 89.8%; NPV, 100.0% vs. 92.7%; accuracy, 100.0% vs. 91.1%, respectively).

**Table 4:** Diagnostic performance of combined washout Tg and CYFRA 21-1 according to gender (Tg ≥ 22.62 ng/ml or CYFRA 21-1 ≥ 2.63 ng/mL).

	Female	Male	*p*-value
n	37	16	
N	74	32	
Sensitivity (%)	92.7	100.0	0.556
Specificity (%)	87.9	94.1	0.650
PPV (%)	90.5	93.8	1.000
NPV (%)	90.6	100.0	0.541
Accuracy (%)	90.5	96.9	0.430

n: number of patients; N: number of LNs.

*Tg* thyroglobulin, *PPV* positive predictive value, *NPV* negative predictive value.

**Table 5:** Diagnostic performance of combined washout Tg and CYFRA 21-1 according to age status (Tg ≥ 22.62 ng/ml or CYFRA 21-1 ≥ 2.63 ng/mL).

	Age < 55 years	Age ≥ 55 years	*p*-value
n	42	11	
N	84	22	
Sensitivity (%)	97.8	81.8	0.104
Specificity (%)	92.3	81.8	0.301
PPV (%)	93.6	81.8	0.237
NPV (%)	97.3	81.8	0.127
Accuracy (%)	95.2	81.8	0.056

n: number of patients; N: number of LNs.

*Tg* thyroglobulin, *PPV* positive predictive value, *NPV* negative predictive value.

**Table 6:** Diagnostic performance of combined washout Tg and CYFRA 21-1 according to the aggressiveness of histology (Tg ≥ 22.62 ng/ml or CYFRA 21-1 ≥ 2.63 ng/mL).

	Non-aggressive histology	Aggressive histology	*p*-value
n	45	8	
N	90	16	
Sensitivity (%)	93.6	100.0	1.000
Specificity (%)	88.4	100.0	1.000
PPV (%)	89.8	100.0	1.000
NPV (%)	92.7	100.0	1.000
Accuracy (%)	91.1	100.0	0.604

n: number of patients; N: number of LNs. Non-aggressive histology: classic, follicular variant. Aggressive histology: tall cell variant, diffuse sclerosing variant.

*Tg* thyroglobulin, *PPV* positive predictive value, *NPV* negative predictive value.

## Discussion

This study is the first prospective work to accurately align preoperative confirmed LNs through needle localization and perform FNA to assess washout Tg and CYFRA 21-1 levels intraoperatively. We demonstrated significant differences in both Tg and CYFRA 21-1 levels between metastatic and benign LNs in thyroid cancer patients. The conjunction of Tg and CYFRA 21-1 showed the best diagnostic performance with better sensitivity, specificity, PPV, NPV, and accuracy compared to using Tg or CYFRA 21-1 alone. In addition, our results suggest that combining Tg and CYFRA 21-1 improves assay efficiency in patients with aggressive features such as male sex, younger age, and aggressive histology, despite lacking statistical significance.

In a prior investigation conducted at our institution, it was found that the washout CYFRA 21-1 level improved the diagnostic capability of identifying metastatic cervical LNs in patients with thyroid cancer, when combined with FNAC and washout Tg level^27^. The key difference in the present work was the precise matching of preoperatively confirmed metastatic LNs with those evaluated during surgery; this enabled more accurate interpretation of the final pathologies and the washout Tg and CYFRA 21-1 levels. Many studies have suggested that the CYFRA 21-1 level is a useful prognostic factor in patients with various cancers, but most have only measured serum levels of this marker^21,22,30,31^. The washout CYFRA 21-1 levels of LN FNA samples have seldom been assayed; no useful diagnostic/prognostic cutoff has yet been established. Here, the optimal cut-off indicated by the ROC curve was 2.63 ng/mL. Lee et al. showed that the conjunction of FNAC with the washout CYFRA21-1 and Tg levels afforded outstanding diagnostic performance, with a sensitivity of 98.8%, a specificity of 93.1%, a PPV of 98.8%, an NPV of 88.2%, and an accuracy of 90.0% when the washout CYFRA 21-1 cutoff was 1.1 ng/mL^27^. In comparison, although our cutoff was higher, our NPV and accuracy were better. This may be explained by the fact that we included only patients with confirmed lateral metastases, which may have resulted in higher concentration levels compared to previous study participants. Another potential reason is that we assessed intraoperative washout CYFRA 21-1 levels rather than conducting preoperative measurements, as done in Lee et al.’s previous study. The differences in sampling timing and methods could account for the variations in values.

While research on washout CYFRA 21-1 obtained through FNA in thyroid cancer is limited, studies in other cancers^36–38^ have provided valuable insights. For instance, in breast cancer, where axillary LN metastasis is prevalent, the extent of surgery varies significantly depending on the presence of axillary LN metastasis. Therefore, it is crucial not to miss this distinction. Choi et al. demonstrated significantly elevated washout CYFRA 21-1 levels in metastatic LNs compared to benign LNs (*p* = 0.001) and proposed that assessing washout CYFRA 21-1 through a US-FNA specimen could yield diagnostic accuracy comparable to cytology^36^. In an investigation exploring FNA tumor markers in cervical lymphadenopathy originating from known malignancies, it was revealed that washout CYFRA 21-1 levels were significantly higher in the metastatic LNs from lung cancers compared to benign LNs (p = 0.003)^39^. These findings underscore the potential utility of washout CYFRA 21-1 assessment in different cancers, emphasizing its diagnostic significance.

Several studies have suggested that serum levels of CYFRA 21-1 are prognostic of thyroid cancer progression^21,22^. Jeong et al. demonstrated the utility of such measurements in thyroid cancer patients with distant metastases who exhibited disease progression^22^. In the study, measurement of serum CYFRA 21-1 emerged as a valuable substitute biomarker for tumor advancement, particularly in instances where Tg remains undetectable or unmonitored due to the presence of Tg antibodies (TgAb) or in poorly differentiated thyroid carcinoma. In addition to washout Tg and CYFRA 21-1, incorporating serum CYFRA 21-1 can be more useful in distinguishing recurrence when interpreting serum Tg levels becomes challenging due to the presence of TgAb after surgery. Approximately 25% of individuals who have undergone initial surgery for DTC exhibit TgAb, which can potentially interfere with precise measurements of Tg^40^. Therefore, the integration of serum and washout CYFRA 21-1 may aid in evaluating the disease status both before and after surgery, not only in identifying local recurrence but also in determining the diagnosis and treatment decisions for distant metastasis, especially for patients with undifferentiated cancers.

In this study, the Tg cutoff value obtained through ROC curve analysis was 22.62 ng/mL. This is very close to those of the literature; the data are consistent. Pacini et al. used a cutoff of 21.7 ng/mL to distinguish benign from malignant LNs^41^; Salmashõglu et al. employed 28.5 ng/mL^16^. However, the cutoff range for washout Tg varies from 0.9 to 50 ng/mL; there is as yet no standard criterion as no consensus has been attained^11,13,15,20,41–43^. In practice, cases whose Tg levels are low but FNAC indicates malignancy, or whose Tg levels are high but FNAC does not suggest malignancy, are not uncommon. In the study conducted by Lee et al., the discordance rate between FNAC and washout Tg was 19.6% in metastatic lymph nodes and 13.0% in benign lymph nodes^44^. In such cases, and in those for whom cytology and the Tg assay are negative but US suggests malignancy, washout CYFRA 21-1 assay might reduce the false-negative level and identify the LNs that require resection.

Another distinctive aspect of this study is that preoperative hook-needle localization was performed in all patients. In the context of thyroid cancer surgery, the adoption of US-guided LN needle localization represents a significant advancement^33–35,45,46^. This approach, reliant on US imaging, presents a range of substantial advantages. Firstly, it ensures the precise identification and localization of critical LNs, a crucial aspect during LN excision procedures^45^. Furthermore, it enhances surgical safety significantly by furnishing surgeons with precise LN coordinates, thereby reducing the risk of unintended damage to surrounding vital structures. The precision it offers in surgical techniques plays a vital role in minimizing tissue trauma^33^. Moreover, this method expedites surgical procedures, leading to reduced overall surgical duration and, consequently, shorter patient recovery times. Equally noteworthy is its capacity to distinguish the benign or malignant nature of tumors with increased accuracy, facilitating more informed surgical planning and guiding comprehensive treatment strategies for patients. In summary, the application of US-guided LN hook-needle localization holds great potential not only for enhancing surgical precision and safety in thyroid cancer procedures but also for optimizing treatment outcomes.

There are various clinical features indicative of an aggressive course in DTC. Notably, characteristics such as male sex, young age, and aggressive variants have been extensively documented in previous literature as exhibiting aggressive features such as LN metastases or being associated with poorer prognoses^47–50^. In this study, we revealed that the combined washout Tg and CYFRA 21-1 assay presents enhanced diagnostic performance in populations characterized by male gender, younger age, and aggressive histological variants of DTC, despite lacking statistical significance. In particular, in the age-based groups, the diagnostic accuracy was higher in individuals under 55 compared to those aged 55 and above, with a p-value of 0.056. This value, being close to 0.05, is interpreted as indicating a significant result. This suggests that this combined assay may offer a significant improvement over Tg alone in terms of diagnostic accuracy, particularly in patient subgroups where a more aggressive clinical course of DTC is anticipated. Given these findings, the assay has potential as a critical diagnostic tool for early and accurate identification of high-risk DTC patients, enabling timely and targeted therapeutic interventions.

In the present study, several subanalyses yielded intriguing outcomes. When comparing the efficacy of this diagnostic method according to BRAF^V600E^ mutation status, a tendency for superior diagnostic performance was observed in BRAF^V600E^-negative cases compared to those that were BRAF^V600E^-positive (supplementary Table S1). Given that the prevalence of BRAF^V600E^ positivity in papillary thyroid carcinoma in Korea typically reaches 80–85%^51,52^, this diagnostic method may prove particularly useful in the minority of cases that are BRAF^V600E^-negative yet suspected of having metastatic lymph nodes. Additionally, we analyzed the correlation between LN size and washout Tg and CYFRA 21-1 levels. Our Pearson correlation analysis revealed no significant association between washout Tg levels and LN size (r = − 0.012, *p* = 0.929), aligning with several previous studies that found similar results^53,54^. However, a positive correlation was found between CYFRA 21-1 levels and LN size (r = 0.294, *p* = 0.028) (Supplementary Fig. S1). However, due to the small sample size, there were only four BRAF^V600E^-negative patients, from whom a total of eight lymph nodes were harvested. This limitation may affect the reliability of the aforementioned analysis results. Future research with a larger sample size, encompassing a broader range of patients and including main genetic alterations like BRAF^V600E^, RAS, and RET, could yield further intriguing findings in this area.

This study had several strengths. First, it was prospective in nature and all washout Tg and CYFRA 21-1 samples were collected by a single surgeon, thus reducing selection bias and observer bias. Second, preoperative localization allowed accurate sampling of all identified LNs across the entire cohort. However, there were several limitations. First, the sample size was relatively small and the study was confined to one institution. Second, all patients had lateral cervical LN metastases, thus advanced thyroid cancer; selection bias may have been in play.

In the future, studies with larger thyroid cancer populations, that evaluate other possible biomarkers, and that explore the associations between biomarker levels and clinical indicators would be valuable.

In conclusion, the combined utilization of both washout Tg and CYFRA 21-1 levels affords a diagnostic efficacy superior to those of the individual levels. The washout CYFRA 21-1 assay may be useful when a malignant LN is suspected, but the FNAC and washout Tg level are ambiguous or disagree in diagnostic terms. A larger prospective study with more thyroid cancer patients would establish a strong washout CYFRA 21-1 cutoff criterion.

## Materials and methods

### Patients

In this prospective study, 53 patients ranging in age from 16 to under 75 years were recruited. They underwent thyroid surgery to treat DTCs with lateral cervical LN metastases at Seoul St. Mary’s Hospital (Seoul, Korea) from May 2020 to May 2021. The inclusion criteria were patients diagnosed with thyroid cancer and lateral cervical LN metastases identified via preoperative FNAC who were scheduled for thyroidectomy with lateral neck dissection, and those scheduled for lateral neck dissection to treat cervical LN recurrences after thyroid cancer surgery who evidenced suspected LN metastases in neck US. The exclusion criteria included cases where preoperative LN FNAC did not indicate metastasis of DTC, patients under 16 or over 75 years of age, and those with distant metastases of DTC or poor overall condition. Individuals meeting these criteria were excluded from the study. No case was lost to follow-up. The age criterion for subanalysis was set at 55 years, in line with the age-specific threshold of the current 8th edition of the AJCC TNM staging system^55^. This study adhered to all relevant tenets of the Declaration of Helsinki (as revised in 2013) and was approved by the Institutional Review Board of Seoul St. Mary’s Hospital, Catholic University of Korea (IRB no. KC20OISI0316). We obtained written informed consent from all patients after they received comprehensive information.

### Lymph node selection

LNs for Tg and CYFRA 21-1 measurement were determined to be those diagnosed with DTC metastasis based on preoperative US and FNAC. The suspicion of malignancy on US was assessed based on a combination of factors such as hypoechogenicity, hyperechoic punctuation, cystic appearance, microcalcification, and loss of fatty hilum^56^. In cases where multiple LNs were suspected to be metastatic, the largest suspicious LN was selected, and its malignancy was ultimately confirmed by FNAC. Benign LNs, identified as being adjacent to the confirmed malignant LNs, were selected and harvested during surgery. This selection was based on their benign appearance, characterized by regular margins, oval shape, and the presence of a fatty hilum, as confirmed on preoperative US.

### Surgical extent and aspiration of lymph node

The initial operation featured total thyroidectomy with cervical dissection; if recurrence developed, reoperation was confined to cervical dissection. During surgery, CYFRA 21-1 levels were assayed in individual LNs using the following method:

1. Prior to surgery, the hook-needle localizations identified LNs with confirmed metastases by FNAC.
2. During surgery, the identified LNs were excised.
3. During surgery, adjacent benign LNs were also excised.
4. During surgery, two aspirations per LN were performed using 22-gauge needles.
5. Washout Tg levels and CYFRA 21-1 levels were measured in both specimens by needle aspiration.
6. After surgery, permanent pathological diagnosis was conducted.

### Washout Tg and CYFRA 21-1 measurement

Tg levels were determined using a monoclonal antibody immunoradiometric assay (Cisbio Bioassays, Codolet, France). Washout CYFRA 21-1 levels were measured using an immunoradiometric assay kit (ELISA-CYFRA, Cisbio Bioassay, Codolet, France). The samples underwent a triple wash process, with the final radioactivity measured using a Gamma counter, the detection limit being 0.05 ng/mL. The established reference range for CYFRA 21-1 was 0.0–3.6 ng/mL.

### Identification of BRAF^V600E^ mutation presence

The presence of the B*RAF*^*V600E*^ mutation was tested in 52 patients*.* Genomic DNA was extracted from formalin-fixed paraffin-embedded tissue sections, each 10 μm thick. Under the guidance of a microscope, tumor areas were precisely separated using a scalpel. The examination focused on the BRAF gene's exon15, specifically at codon600 (c.1799), referencing the sequence NM_004333.4^57^. This analysis was conducted using the PNAClamp™ BRAF Mutation Detection Kit by Panagene, based in Daejun, South Korea^58,59^. The test's sensitivity might be reduced in cases with minimal tumor tissue or where mutation frequency is under 1%. Quality control of the reagents used in this test was thoroughly verified, and pathologists who are certified confirmed the results.

### Primary and secondary endpoints

The primary endpoints were the differences in CYFRA 21-1 levels and Tg between metastatic and benign LNs. The secondary endpoints were the final pathologic diagnoses, and the concentrations of washout CYFRA 21-1 and Tg in individual LNs.

### Statistical analysis

Continuous variables were represented using mean values along with standard deviations, while categorical variables were expressed as counts and corresponding percentages. To compare continuous variables, Student’s t-test was employed. The cutoff values for washout Tg and CYFRA 21-1 levels were determined through the analysis of the AUC derived from ROC curve analysis. We evaluated diagnostic performance metrics, including sensitivity, specificity, PPV, NPV, and accuracy. Statistical significance was defined as *p* < 0.05. Statistical analysis was conducted using IBM Statistical Package for the Social Sciences (version 24.0; IBM Corp., Armonk, NY, USA).

### Institutional review board statement

This study was conducted in accordance with the Declaration of Helsinki, and approved by the Institutional Review Board of Seoul St. Mary’s Hospital, The Catholic University of Korea (IRB No: KC20OISI0316 and date of approval: 2020.07.24).

### Informed consent

Written informed consent explaining the purpose and procedures has been obtained from all eligible subjects by clinician.

### Supplementary Information

Supplementary Figure S1.

Supplementary Table S1.

## Data Availability

The data are not publicly available due to privacy or ethical restrictions. Further enquiries can be directed to the corresponding author.
