# Supplementary figures and images for: Diagnostic utilities of washout CYFRA 21-1 combined with washout thyroglobulin for metastatic lymph nodes in thyroid cancer: a prospective study
Source: Sci Rep. 2024 Mar 31;14:7599. doi: 10.1038/s41598-024-58093-9 (PMC10982287; doi:10.1038/s41598-024-58093-9)

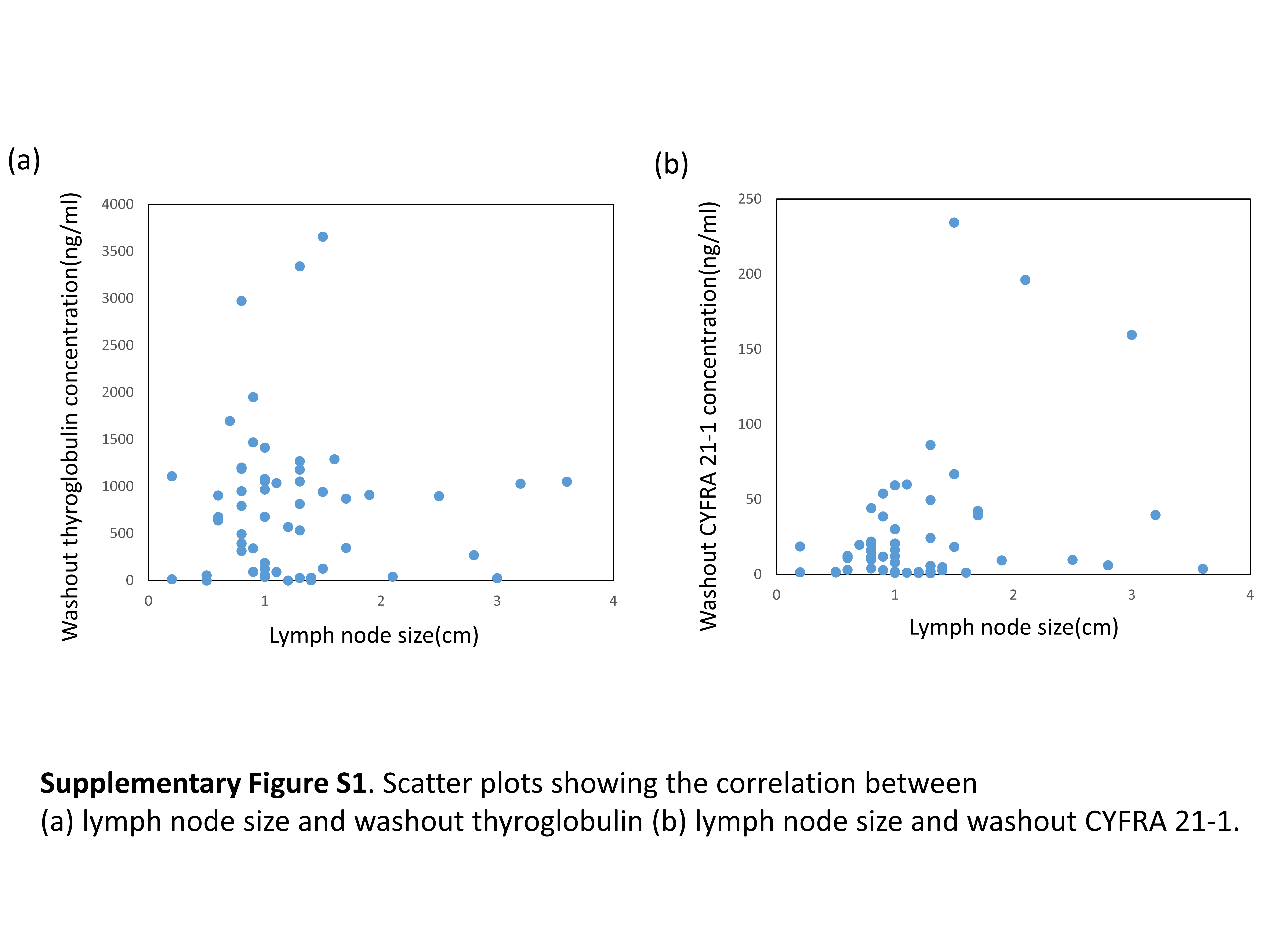

Supplement: Supplementary file 1 — Supplementary Figure S1. [file 41598_2024_58093_MOESM1_ESM.tif]
