# Supplementary material for: Diagnostic utilities of washout CYFRA 21-1 combined with washout thyroglobulin for metastatic lymph nodes in thyroid cancer: a prospective study
Source: Sci Rep. 2024 Mar 31;14:7599. doi: 10.1038/s41598-024-58093-9 (PMC10982287; doi:10.1038/s41598-024-58093-9)
Supplement: Supplementary file 2 — Supplementary Table S1. [file 41598_2024_58093_MOESM2_ESM.docx]

|  | **n** | **N** | **Sensitivity**  **(%)** | **Specificity**  **(%)** | **PPV (%)** | **NPV (%)** | **Accuracy**  **(%)** |
| --- | --- | --- | --- | --- | --- | --- | --- |
| **BRAF^V600E^ positivity** | 48 | 96 | 96.1 | 88.9 | 90.7 | 95.2 | 92.7 |
| **BRAF^V600E^ negativity** | 4 | 8 | 100.0 | 100.0 | 100.0 | 100.0 | 100.0 |

**Supplementary Table S1.** Diagnostic performance of combined washout Tg and CYFRA 21-1 according to BRAF^V600E^ mutation status (Tg ≥ 22.62 ng/ml or CYFRA 21-1 ≥ 2.63 ng/mL).

n: number of patients; N: number of LNs.
Abbreviation: BRAF^V600E^, B-Rapidly Accelerated Fibrosarcoma gene V600E; Tg, thyroglobulin; PPV, positive predictive value; NPV, negative predictive value.
